# Limitations of soil-applied non-microbial and microbial biostimulants in enhancing soil P turnover and recycled P fertilizer utilization - a study with and without plants

**DOI:** 10.3389/fpls.2024.1465537

**Published:** 2024-11-12

**Authors:** Michelle Natalie Herrmann, Lydia Grace Griffin, Rebecca John, Sergio F. Mosquera-Rodríguez, Peteh Mehdi Nkebiwe, Xinping Chen, Huaiyu Yang, Torsten Müller

**Affiliations:** ^1^ Institute of Crop Science, Department of Fertilization and Soil Matter Dynamics, University of Hohenheim, Stuttgart, Germany; ^2^ College of Agricultural and Environmental Sciences, University of Georgia Athens, Athens, GA, United States; ^3^ Institute of Biology, University of Hohenheim, Stuttgart, Germany; ^4^ Institute of Plant Physiology and Biochemistry, University of Hohenheim, Stuttgart, Germany; ^5^ College of Resources and Environment, Academy of Agricultural Sciences, Southwest University, Chongqing, China; ^6^ Interdisciplinary Research Center for Agriculture Green Development in Yangtze River Basin, Southwest University, Chongqing, China

**Keywords:** biostimulants, biofertilizers, P availability, P turnover, phosphorus solubilizing bacteria, recycled fertilizers, struvite, sewage sludge ash

## Abstract

**Introduction:**

Phosphorus recovery from waste streams is a global concern due to open nutrient cycles. However, the reliability and efficiency of recycled P fertilizers are often low. Biostimulants (BS), as a potential enhancer of P availability in soil, could help to overcome current barriers using recycled P fertilizers. For this, a deeper understanding of the influence of BSs on soil P turnover and the interaction of BSs with plants is needed.

**Methods:**

We conducted an incubation and a pot trial with maize in which we testednon-microbial (humic acids and plant extracts) and microbial BSs (microbial consortia) in combination with two recycled fertilizers for their impact on soil P turnover, plant available P, and plant growth.

**Results and discussion:**

BSs could not stimulate P turnover processes (phosphatase activity, microbial biomass P) and had a minor impact on calcium acetate-lactate extractable P (CAL-P) in the incubation trial. Even though stimulation of microbial P turnover by the microbial consortium and humic acids in combination with the sewage sludge ash could be identified in the plant trial with maize, this was not reflected in the plant performance and soil P turnover processes. Concerning the recycled P fertilizers, the CAL-P content in soil was not a reliable predictor of plant performance with both products resulting in competitive plant growth and P uptake. While this study questions the reliability of BSs, it also highlights the necessity toimprove our understanding and distinguish the mechanisms of P mobilization in soil and the stimulation of plant P acquisition to optimize future usage.

## 1 Introduction

Innovative solutions are needed to close the global gap between the intensive use of finite P fertilizers, and open P cycles on the other hand resulting in environmental damage (Carpenter and Bennett, 2011; Müller and Zhang, 2019; Jasinski, 2020; Panagos et al., 2022). So far, the usage of P from recycling streams has not yet reached its full potential, but the full recovery of P from streams will be critical for the environment and for fertilizer production in the future. In Austria, for example, it was estimated that 70% of the currently applied fertilizers could be substituted by P from alternative sources, while at the moment only 27% of the P from wastewater is being recycled (Egle et al., 2014). However, many recycled P fertilizers, particularly incineration products, are characterized by a lower P availability for plants leading to a lower P use efficiency compared to conventional synthetic P fertilizers such as triple super phosphate (Roberts and Johnston, 2015; Kratz et al, 2019). Even though a thermo-chemical treatment of sewage sludge ashes with alkaline additives was able to increase the P availability in soil and improved P related plant responses (Severin et al., 2014) the gap of the effectiveness of these products compared to readily available P of conventional synthetic fertilizers is still pronounced (Nanzer et al., 2014). Precipitation products such as struvite-based fertilizers can keep up with conventional synthetic fertilizers in terms of P bioavailability, but have shown a considerably variable performance in pot and field experiments (Römer and Steingrobe, 2018) which makes them unattractive for farmers (Utai et al., 2022). Therefore, strategies on how to increase the performance, reliability and finally the acceptance of these recycled P products need to be explored.

The combination with biostimulants (BS), also known as bioeffector, could serve as one such promising approach. Over the past decade, BSs have attracted attention as a potential way to improve the sustainability of current food production through the reduction of the demand of inputs like fertilizers (Povero et al., 2016; Rouphael and Colla, 2020). The application of BSs targets to increase plant growth by enhancing nutrient acquisition, increasing stress tolerance, or crop quality without the need for additional nutrients or pesticides to the system (Du Jardin, 2015). The potential positive impact on yield and nutrient uptake of BSs was demonstrated in several meta-studies (Schütz et al., 2017; Herrmann et al., 2022; Li et al., 2022). The combination of recycled P fertilizers and BSs increased the reliability and effectiveness of new, alternative P fertilizer products (sewage sludge ashes and struvites), as studies have found positive effects of this approach on yield and P uptake (Thonar et al., 2017). However, reliability remains a crucial factor since other studies could not confirm the impact on P use efficiency (Wollmann et al., 2018; Jastrzębska et al., 2019). In order to optimize the functionality of BS products in combination with recycled fertilizers, a deeper understanding of the mechanisms behind the BS application in the interface of soil and plants is necessary. There is limited knowledge regarding the key drivers of P dynamics and the specific processes involved– the direct P mobilization from the soil, and the stimulation of native soil microorganisms, or the modification of plant responses for P uptake.

BSs can be grouped into two categories: microorganisms - single strains or mixtures of several strains-, and natural-active compounds, which alter soil characteristics, shape soil microbial community, and P availability in different manners. BS products based on microorganisms may directly mobilize P in the soil or stimulate the plant’s mechanisms for P acquisition. An enormous diversity of microorganisms exists in natural soil populations which are capable of solubilizing P from less plant accessible sources, such as P bound to clay particles (Kalayu, 2019). By the exudation of organic anions (carboxylates) and other complexing/chelating compounds, extra-cellular enzymes, and the reduction of the pH, P solubilizing microorganisms can degrade organic P compounds and enable the release of P from less bioavailable P pools (Rawat et al., 2021).

Regarding the stimulation of plant’s nutrient uptake, many plant growth-promoting microorganisms interfere with root morphology by shifting the hormone balance in plants. Several plant growth-promoting microorganisms produce phytohormones such as auxin. Others release compounds that interfere with hormone production in the plant which may lead to a higher root growth and increased root branching (Vacheron et al., 2013) and thereby, ameliorate the plant’s nutrient acquisition potential.

The pairing of microorganisms based on their mode of action was proposed as a promising possibility to exploit synergies (Santoyo et al., 2021). One prominent example is mycorrhiza helper bacteria, which support the symbiosis between arbuscular mycorrhizal fungi (AMF) and the plant, while simultaneously promoting the nutrient uptake of the plant by mobilizing P from the soils and/or stimulating root proliferation which is also advantageous for the colonization of AMF (Frey-Klett et al., 2007; Hameeda et al., 2007; Sangwan and Prasanna, 2022).

In addition to microbial products, a wide variety of naturally active compounds are used and sold as BSs such as extracts from seaweed and other plants, as well as humic and fulvic acids. These BSs may also have an impact on the nutrient uptake by plants and plant performance under nutrient limiting conditions. Humic acids could possibly increase the amount of plant available P forms in the soil by complexing iron (Gerke, 1993). Extracts often contain phytohormones which, similar to plant growth promoting microorganisms, can stimulate root growth (Mukherjee and patel, 2020). Additionally, these non-microbial BSs can serve as a carbon source for microbes in the soil. Previous studies have shown an increase in respiration and soil microorganism diversity when extracts were applied (Alam et al., 2013; Hussain et al., 2021; Prisa, 2021). Shukla and Prithiviraj (2020) also demonstrated an influence on gene expression related to P starvation response which could result in higher P uptake by the plant when seaweed extracts were applied to maize under P limiting conditions.

However, there is insufficient evidence regarding which of the above-mentioned mechanisms are decisive for the observed impact on plant performance. Furthermore, it is not clear, whether BSs only increase the plant performance by an interaction with the plant or, as often suggested, change the P turnover in soil and thereby, increase the P availability in the soil. In particular, non-microbial products are often neglected in studies about potential impacts on soil and soil microbial activity by BS products and direct comparisons of non-microbial and microbial BSs are lacking. For example, Li et al. (2022) claim the impact of the soil is a major determinant for the improvement of plant performance. Higher responses were observed when these products were applied to the soil compared to a foliar application (Li et al., 2022). In addition, little is known if BSs not only can improve P availability in soil, but if they can increase the P fertilizer use efficiency of recycled P fertilizers by the same mechanisms.

The purpose of our study was to address the impact of BSs on soil P mobilization, the comparison of different BSs on their effectiveness to alter soil P processes, and their potential benefits in the utilization of recycled fertilizers. We applied BSs directly to the soil in combination with recycled fertilizers to assess their impact on P turnover and plant growth. To distinguish between pure soil-based effects and effects related to growing maize, corresponding incubation, and greenhouse pot experiments were performed. Our hypotheses are as follows: (1) BSs increase the P availability in soil in a system without plants. Thereby, microbial BSs directly act in the soil by stimulation taking advantage of their own P mobilization pathways in soil. Non-microbial BSs can induce similar changes in the soil by stimulating soil P turnover processes by native soil microorganisms. (2) Through the addition of P by the application of recycled fertilizers, mobilization pathways of soil microorganisms are stimulated, and a positive interaction of BSs and recycled fertilizers is anticipated. (3) In the greenhouse trial, BSs increase plant growth and the P acquisition of plants under P limited conditions. The stimulatory impact of BSs on the P cycling in soil amplifies as products are designed to interact with the plants and additionally, root growth is promoted. (4) In terms of the application of recycled fertilizers, BSs can improve the P fertilizer use efficiency. Synergies of a combination of recycled fertilizers and BSs are more pronounced in an environment with plants than without.

## 2 Materials and methods

### 2.1 Treatments

In this experiment, two recycled fertilizers were chosen due to promising results in earlier studies: one was a struvite containing P fertilizer (0.66% N, 6.98% total-P of which< 1% is water soluble, 0.24% K, STR) produced from biogas digestate with a new procedure called NuTriSep, the second one was a thermic-chemically treated sewage sludge-ash (7.97% total-P, 5.9% Fe, 5.1% Al, SSA) from the ASH -DEC procedure (Hermann and Schaaf, 2016). The NuTriSep procedure separates P, N, and K from the digestate to enable the application of individual nutrients orientated on actual crop demand. We tested in this trial the P recyclate. Both soils received the same amount of P (50 mg P kg^-1^ dry substrate). The recycled fertilizers did not apply a considerable amount of N or K, that is why the fertilizer amount of N and K was not adopted.

Regarding the BSs, we used commercial products or products that will soon be released to the local market to increase the chance of a successful inoculation of the microbial BSs and an effective action in the soil. We have chosen to include non-microbial and microbial biostimulants to be able to directly compare their effectiveness. For the microbial biostimulants, we only included microbial consortia as they were shown to be more effective than single strains (Herrmann et al., 2022). Therefore, we included two different plant extracts, one humic acid, and two microbial consortia. The available details of the products as well as the applied dosages can be found in **Table 1**. The microbial products contained microorganisms with known P solubilization ability (Elhaissoufi et al., 2022). To ensure an effective and visible action of these products, we used a higher dosage than the recommendation of the manufacturers, which may have influenced the results of the trial. For the microbial consortia inoculation, the application rate of both BSs was adopted to reach the same colony-forming unit per gram soil which was set to 10^8^ colony-forming units (cfu) g^-1^ soil as successfully implemented in earlier experiments (Nkebiwe et al., 2016). This corresponded to a 100 times higher dosage compared to the recommendation of the manufacturer. To make the results comparable between non-microbial and microbial products, we also chose for the incubation trial without plants for the non-microbial products a 100 times higher dosage than the recommendation of the manufacturer. However, because the plant extracts may become phytotoxic (manufacturers advice) if the applied concentrations are too high, we reduced the application amount of the non-microbial BSs to a 10 times higher dosage compared to the recommendation of the manufacturer in the pot trial which included plants. Nutrient additions due to the low application amount are negligible for the BSs.

**Table 1:** Biostimulants (BS) used in this experiment and dosages applied.

Product	Company	Ingredient	Trial	Dosage
**Plant extract 1 (AE1)**	BioAtlantis Ltd., Ireland	Seaweed-based blends with addition of botanical extracts	Incubation/pot trial	0.21 ml kg^-1^ soil: 100x producer’s recommendation (incubation) 0.02 ml kg^-1^ soil: 10x producer’s recommendation (pot)
**Plant extract 2 (AE2)**	BioAtlantis Ltd., Ireland	Seaweed-based blends with addition of botanical extracts	Incubation trial	100x producer’s recommendation
**Humic acid (HA)**	Humintech	14% Humic acid 4% Fulvic acid	Incubation/pot trial	5 ml kg^-1^ soil: 100x producer’s recommendation (incubation) 0.5 ml kg^-1^ soil: 10x producer’s recommendation (pot)
**Microbial Consortia (M1)**	Sourcon Padena	Pseudomonas brassicacearum: 2x10^10^ cfu/g Bacillus amyloliquefaciens: 2x10^10^ cfu/g Trichoderma harzianum: 1x10^8^ cfu/g	Incubation trial	0,01 g kg^-1^ soil: 100x producer’s recommendation
**Microbial Consortia (M2)**	Bactiva	Glomus intraradices 132 spores/g; Azospirillum brasilense, Azotobacter chroococcum, Bacillus megaterium, Pseudomonas fluorescens: 4x10^9^ cfu/g	Incubation/pot trial	0,10 g kg^-1^ soil: 100x producer’s recommendation

In the incubation trial, we tested two soils with different P contents, while in the pot trial we only used the soil with the lower P content. Relevant information of the soils can be found in **Table 2**. Both soils were retrieved from the topsoil (0-20 cm) and were sieved with a ø5 mm-sieve after airdrying.

**Table 2:** Soil characteristics.

	Soil 1 (Class A^1^)	Soil 2 (Class B^1^)
**Location**	Oberer Lindenhof -Research station, University of Hohenheim, Germany 48°47’44.4°”N, 9°30’49.9”E	Hirrlingen, Germany 48°24’25.0”N 8°53’44.1”E
**pH (measured in 0.01 M CaCl_2_)**	4.9	5.7
**CAL-P (mg CAL-P kg^-1^ soil) (Calcium-acetate-lactate-solution)**	9.81	22.09
**VDLUFA-P classes (** Wiesler et al., 2018 **) (Verband Deutscher Landwirtschaftlicher Untersuchungs- und Forschungsanstalten e. V.)**	Class A – strong P-deficiency	Class B – medium P-deficiency
**Corg**	2.2%	1.2%
**Texture**	silty clay loam	Silty clay loam
**Clay content**	32.6%	38.6%

1 Class A refers to a strong P-deficiency and Class B to a medium P-deficiency according to Wiesler et al., 2018

Summarizing the above-mentioned treatments, the incubation trial consisted of 36 treatments (2 soils x 6 BSs including one control x 3 fertilizer treatments including one unfertilized control), and the pot trial included 12 treatments (1 soil x 4 BSs including one control x 3 fertilizer treatment including one unfertilized control). In both cases, a randomized complete block design was used with 5 and 4 blocks for the incubation and pot trial, respectively.

### 2.2 Incubation trial

The incubation trial was set up for 8 weeks in incubation cabinets with a temperature of 20°C. 750 g dry soil was each filled in 1 L glasses and watered with distilled water 1 week before the start of the experiment to reach a water holding capacity of 50% which corresponds to 34% (soil 1) and 28% (soil 2) water content. BSs and fertilizers were given at the same time by applying the necessary amounts and thoroughly mixing the soil. The liquid amount given to each of the treatments was balanced by giving distilled water to treatments receiving a lower amount of liquids. The glasses were covered with a thin plastic layer to avoid water loss but to guarantee aerobic conditions in the glasses at the same time. The water content was checked gravimetrically once per week and water was added accordingly.

Soil samples were collected 5 times during the experiment: 2 days, 1 week, 2 weeks, 4 weeks, and 8 weeks after fertilization. For this, soil was watered and mixed again, and a representative sample was taken. Afterwards, when watering, the weight loss due to the sampling was accounted for. For the analysis of microbial biomass P and phosphatase activity, fresh samples were taken 1 week and 4 weeks after fertilization and were stored at 4°C until further analysis.

### 2.3 Greenhouse trial

The greenhouse trial was set up for 6 weeks in the greenhouse facilities at University of Hohenheim from May 2022 to July 2022. Temperature and relative humidity in the greenhouse were recorded automatically several times during the day and night. The average temperature during the growing period was 26.6°C, and the relative humidity was on average 48.3%. The substrate used for this experiment consisted of 70% of dry low P soil (P-Class A) and 30% of quartz sand (ø 0.6-1.2 mm) on a weight basis. Besides P fertilizers, the substrate was fertilized with 150 mg N (kg dry substrate)^-1^ in the form of Ca(NO_3_)_2_, 200 mg K (kg dry substrate)^-1^ in the form of K_2_SO_4_, and 100 mg Mg (kg dry substrate)^-1^ in the form of MgSO4. All these fertilizers were homogeneously mixed with the soil and 6 kg of the substrate (dry matter) was filled in Mitscherlich-pots.

We used the maize (*Zea mays* L.) cultivar Stabil (KWS SAAT SE & Co. KG, Einbeck, Germany). Three seeds were sown and then thinned to one after emergence. BS were applied to the soil close to the seeds. The initial plan was to apply the BS amount once after sowing (in the seed hole) and again after germination (close to the plant after thinning). Since germination of the first set failed and we had to re-sow the seeds, we decided to double the BS application upon the germination of the second planting. This was to make up for any BS concentration lost when taking out seeds/plants. Throughout the experiment, moisture was kept at 70% water holding capacity, and pots were gravimetrically watered 2 to 3 times per week depending on the weather.

Soil samples were taken 1 day after fertilization, post-emergence (6 days after sowing), at the 3-leaf stage (12 days after sowing), at the 6-8 leaf stage (32 days after sowing), and at final harvest at the 9-leaf stage (48 days after sowing), respectively. During the growing period of maize, soil was taken from the first 10 cm of the pots close to the plants and mixed thoroughly. Fresh soil samples were taken for the analysis of microbial biomass P and phosphatase activity at final harvest, which were stored at 4°C until further analysis.

### 2.4 Measurements

#### 2.4.1 Soil analysis

Microbial Biomass P was estimated by the chloroform fumigation extraction method (Brookes et al., 1982) according to the protocol described by Joergensen et al. (1995). Briefly, three sets of soil samples were analyzed for their Olsen-P concentration. One set was fumigated with chloroform for 24 h at 20°C, the other two sets were not fumigated and one of the two were spiked with a P containing Olsen solution to measure the absorption of released P to the soil. and P concentrations in the extracts were measured photometrically according to Murphy and Riley (1962). Microbial Biomass was calculated based on the following equation:


$$MicBioP=\frac{f-n}{\frac{s-n}{a}}*\frac{1}{k}*\frac{FW}{DW}$$

where f is the P concentration of the fumigated soil, n is the P concentration of the non-spiked, non-fumigated soil, s is the P concentration of the non-fumigated but spiked soil, a is the amount of P which is added as a spike to the soil, k is the constant derived from Brookes et al. (1982) and FW/DW is the conversion from fresh soil to dry soil.

For the incubation trial, microbial biomass P was determined in the samples taken 1 week and 4 weeks after fertilization for the highly P deficient soil, whereas for the pot trial samples retrieved at final harvest were measured for microbial biomass P.

Acidic and alkaline phosphatase activity were determined by the p-nitrophenyl phosphate method of Tabatabai and Bremner (1969) with modifications adapted from Rubio et al. (1990) and Redel et al. (2008). Briefly, 2 ml of 0.115 M nitrophenolphosphate was added to 2 g of fresh soil samples together with the buffer solution setting the pH value (5.5. for acid phosphatase, 8.2 for alkaline phosphatase). Additionally, blanks were included which only received water before the water bath, and omega blanks which received nitrophenol (500 µg ml^-1^). Samples were then placed in a water bath for 1 h. Afterwards, 0.5 M CaCl_2_ was added to stop the enzyme reaction and 2 ml of water was given to the samples to equalize the volume between samples and blanks/omega blanks. The blanks and omega blanks received 2 ml of nitrophenolphosphat. Afterwards, the samples were filtered immediately. 1.2 ml of the supernatant was added into a falcon tube with 13.4 ml water and 0.4 ml 0.5 M NaOH and centrifuged. The nitrophenol content was measured photometrically at a wavelength of 420 nm. Phosphatase activity was then calculated with the following equation:


$$Pase=\frac{S-B}{\frac{O-B}{500}}*\frac{FW}{DW}\;$$

with S being the with nitrophenylphosphat for 1 h incubated sample, B being the blank, in which nitrophenylphosphat was added after the incubation, O being the omega Blank, for which 500 µg nitrophenol was added to the sample during the 1 h incubation and FW/DW being the conversion factor from fresh soil to dry soil.

Additionally, blanks without soil were added to test any interference with the organic matter content in the soil. This was not the case in both soils.

For the incubation trial, phosphatase activity was analyzed for samples retrieved 1 week after fertilization and in the pot trial, samples from the final harvest were analyzed.

Plant available P was determined via the Calcium-acetate-lactate extraction (CAL-P) according to the protocol of the VDLUFA (Schüller, 1969; VDLUFA, 1991). For this, 5 g of soil were mixed with 100 ml CAL solution and shaken for 1.5 h. Afterward, the samples were filtered, and P concentrations were measured photometrically in the filtrate. Prior to the analysis, the soil samples were air dried and sieved with a ø 2 mm-sieve. CAL-P was determined at all sampling times. In the samples from the final harvest, soil pH was measured in a 0.01 M CaCl_2_ solution.

#### 2.4.2 Plant analysis

Throughout the growing period, plant height, leaf area, and stem diameter were measured every other week. After harvest, plants were dried at 60°C, weighed, and milled for nutrient content analysis. P content in the whole plant material was analyzed with the microwave digestion method (VDLUFA, 2011). Roots were carefully washed and stored in a 70% ethanol solution at 4°C. Total root length and fine root length were measured by the WinRhizo Pro V. 2009c (Regent Instruments Inc., Canada) software.

### 2.5 Statistical analysis and illustration

Statistical analysis was implemented with SAS Software (Version 9.4. SAS Institute Inc., Cary, NC, USA). To correct for potential variations within one soil analysis method due to different measuring time points, a control soil was always analyzed together with the soil samples. In the statistical model, the control soil was, then, added as a covariate, and blocks were included as a random effect in order to eliminate that error. Our full linear mixed model for the unrepeated case was as follows:


$$\begin{array}{c} y_{ijkl}=µ+b_{i}+s*x_{ijkl}+\alpha_{j}+\beta_{k}+\gamma_{l}+{\left(\alpha \beta \right)}_{jk}+{\left(\alpha \gamma \right)}_{jl}+{\left(\beta \gamma \right)}_{kl}\\ +{\left(\alpha \beta \gamma \right)}_{jkl}+e_{ijkl} \end{array}$$

where µ is the intercept, b is the random effect of the ith block, s is the linear correction factor based on the control soil, α, β, γ are the effects of jth soil, the kth BS and the lth fertilizer. y_ijkl_ is the mean with a homogeneous and normal distributed residual error e_ijkl_. Because in the pot trial only one soil was used, the parameter vectors for soil and all respective interaction terms were excluded.

The final model was then chosen based on the results of the ANOVA by removing factors with non-significant effects from the model.

In the case of repeated measurements, a time effect was included in the model. To consider the correlation among the observation time points, the best fitting variance-covariance matrix was chosen based on the AIC criterion (Wolfinger, 1993). For CAL-P measurements in the pot, leaf area, and stem diameter, time was considered as a metric variable since a simple polynomial model could be fit which was able to explain the data sufficiently. In any other case, time was included as a categorical variable.

Illustrations of the graphs were implemented with the R package ggplot2 (Wickham, 2016). Fisher’s LSD test of pairwise differences (Fisher, 1936) were performed based on the final reduced model and are displayed with letters in the graphs.

## 3 Results

### 3.1 Soil P turnover processes are irresponsive to biostimulant’s application without plants

**Figure 1:**
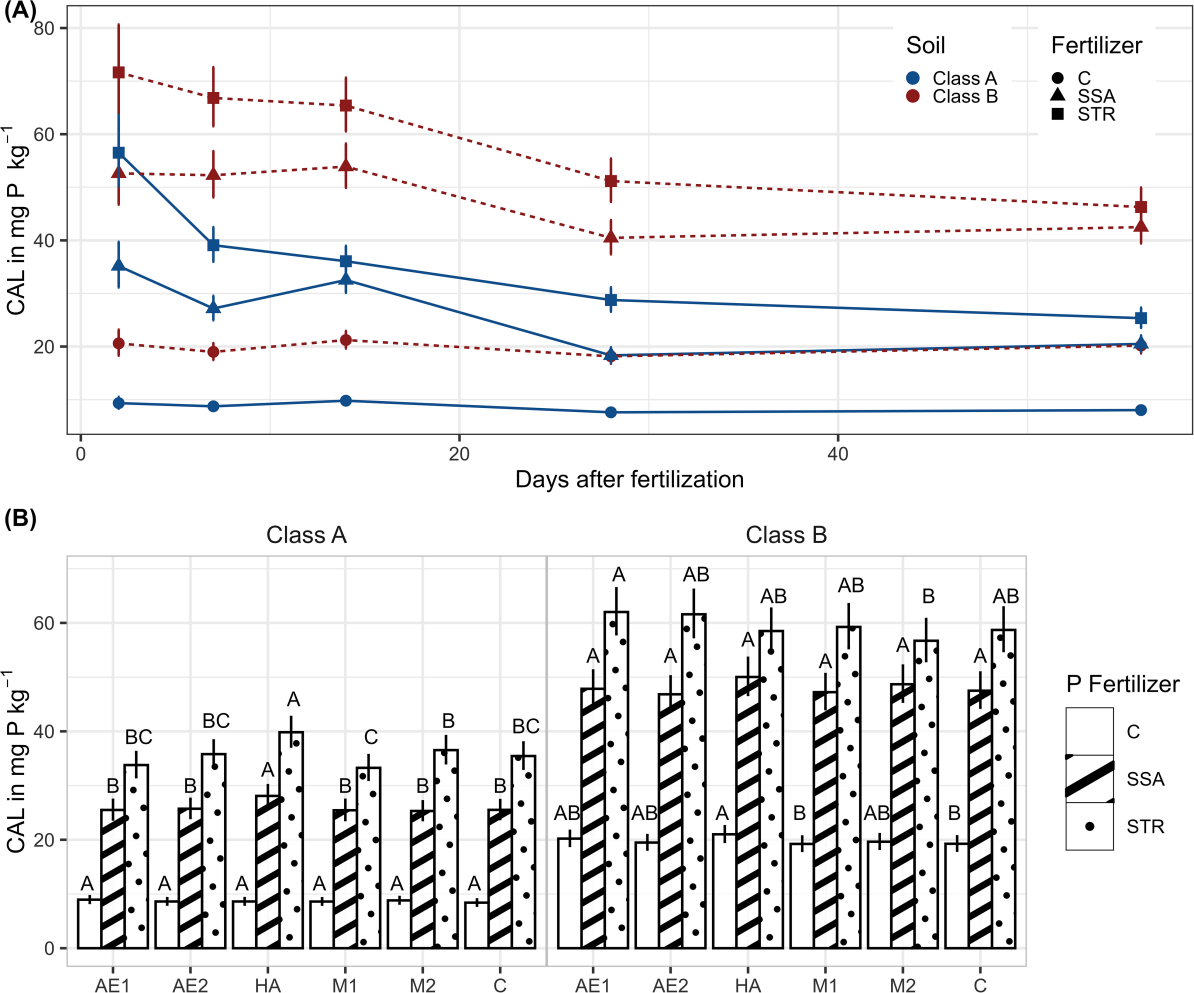
CAL-P in dry soil **(A)** CAL-P in dependence on fertilization, soil and time (n=896). Illustrated are medians of the time points of the measurement with their confidence limits (α=0.05). Statistical differences are not shown for a better visualization. **(B)** CAL-P in dependence on biostimulants and fertilization. Medians with their confidence limits (α=0.05) are depicted (n=896). The letters show the results of the pairwise comparisons of biostimulants within the fertilization (α=0.05). Same letters mean no significant difference between treatments. C, Control; SSA, treated sewage sludge ash; STR, struvite containing P fertilizer; Class A, soil with high P-deficiency; Class B, soil with moderate P-deficiency; AE1 + 2, plant extract; HA, humic acids; M1 + 2, microbial consortia.

In the current incubation test, CAL-P was strongly dependent on soil, fertilizer, BSs, and the sampling time (**Supplementary Table 1**). CAL-P showed a decreasing trend for the fertilizer treatments till the fourth sampling time, 28 days after fertilizer application while the unfertilized control maintained its CAL-P content over time (**Figure 1A**). Thereby, the STR treatment declined faster in the beginning than the SSA leading to a convergence at the end of the experiment of both fertilizer treatments’ CAL-P content in soil. In the case of the moderate P-deficient soil (Class B), the difference between the two fertilizers was no longer significant at the end of the experiment with a content of 42.5 mg (kg dry soil)^-1^ for the SSA treatment and 46.3 mg (kg dry soil)^-1^ for the STR treatment.

The addition of humic acids to the soil resulted in a slightly higher CAL-P content for the unfertilized control in the moderately P deficient soil (Class B), and for the fertilized treatments in the highly P-deficient soil (**Figure 1B**). All other BSs did not vary substantially from the untreated control and the fertilization effect was overall predominant.

**Figure 2:**
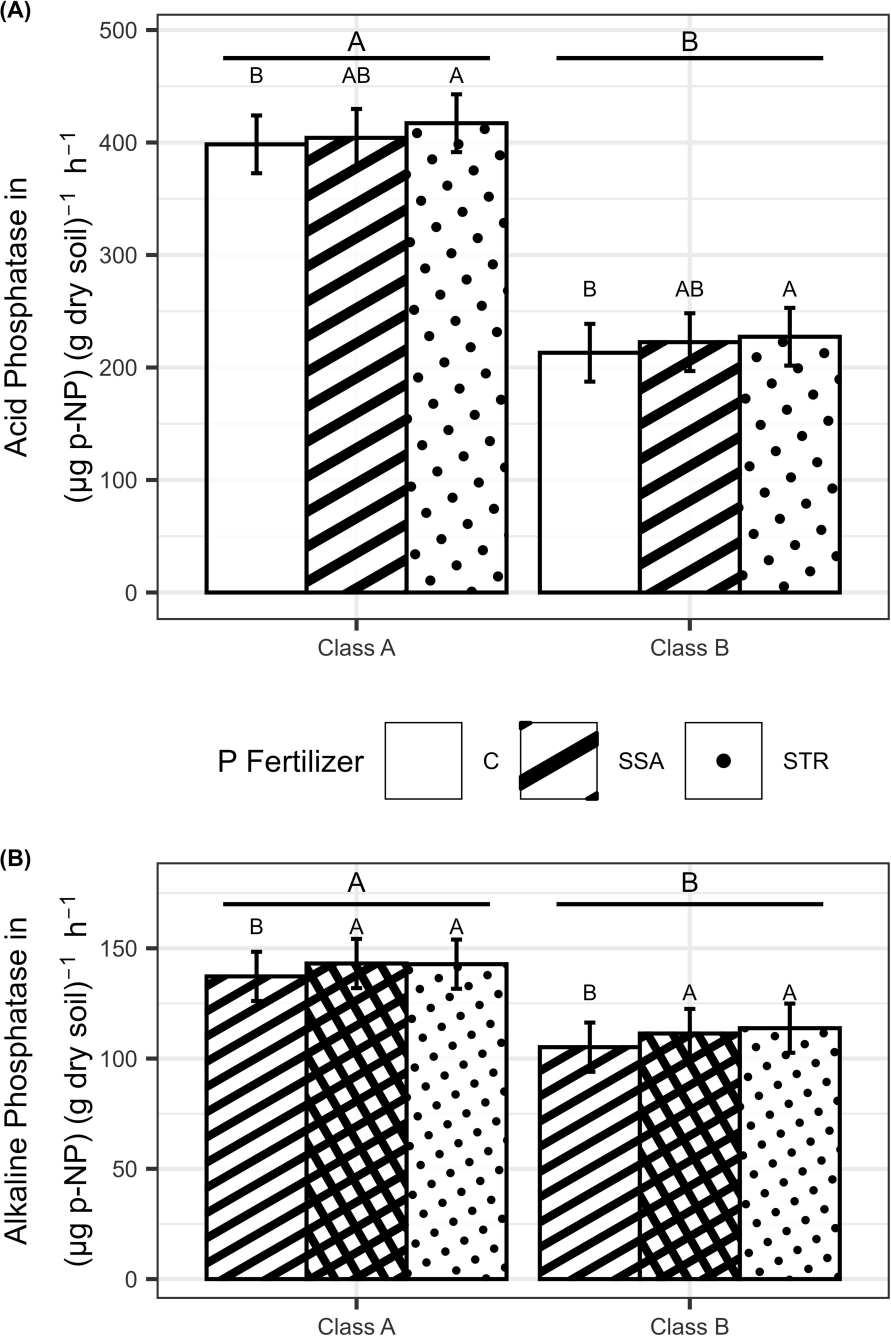
**(A)** Acid Phosphatase activity (n=180) and **(B)** alkaline Phosphatase activity (n=180) in dependence on fertilization and soil. Medians with their confidence limits (α=0.05) are depicted. Same capital letters indicate no significant difference between the fertilization (α=0.05). Letters above the line display the comparison of the soils (α=0.05). C, Control; SSA, treated sewage sludge ash; STR, struvite containing P fertilizer; Class A, highly P-deficient soil; Class B, moderately P-deficient soil.

While the microbial biomass P was not significantly affected by any treatment (**Supplementary Table 1**, data shown in **Supplementary Figure 1**), the acidic and alkaline phosphatase activity were influenced by the fertilizers and the soil (**Supplementary Table 1**). The acidic phosphatase activity expressed as p-nitrophenol development in dry soil over time was increased by 13.4 µg g^-1^ h^-1^ by the struvite containing P fertilizer compared to the unfertilized control independent of the soil (**Figure 2A**). The alkaline phosphatase activity showed a significantly higher activity (by 6.5 µg g^-1^ h^-1^) when fertilized with P (**Figure 2B**). Both enzymes were more stimulated in the soil with the lower P content (**Figures 2A, B**).

The soil pH did not reflect any relevant changes by a BS application or by the fertilizer application which could result in a higher plant P availability (**Supplementary Table 3**).

### 3.2 Biostimulants cannot promote plant growth in P limiting conditions

**Figure 3:**
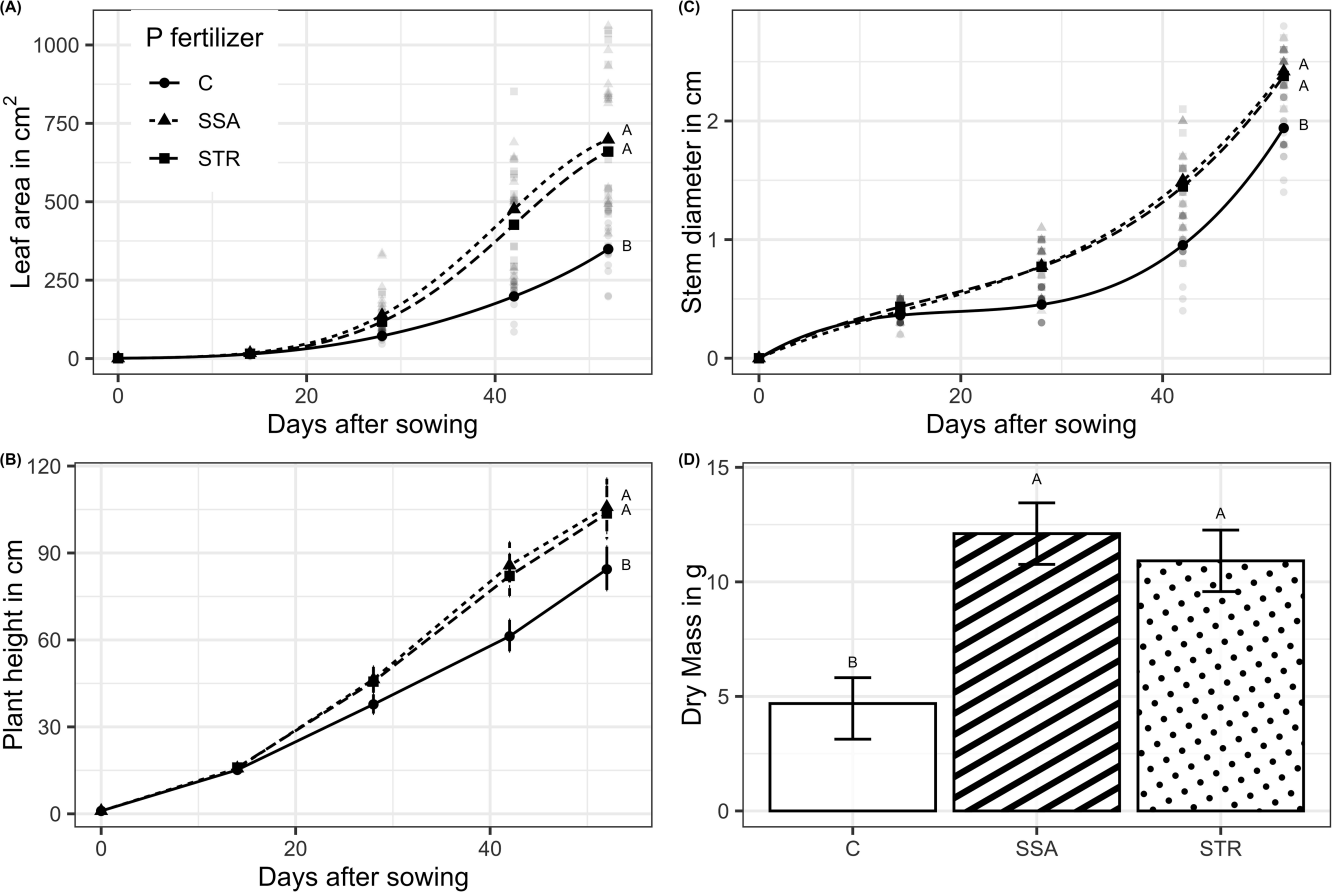
Plant growth traits. **(A)** Leaf area and **(C)** stem diameter as a function of time for the fertilization (n=192). Points represent the estimate from the statistical model at the respective measuring time point while the more transparent dots are raw measurements. **(B)** Plant height in dependence on fertilization and time (n=192). In **(B)**, medians are displayed by dots with their respective confidence limits (α=0.05). **(D)** Dry mass at harvest in dependence on fertilization (n=48). Means are displayed by bars with their confidence limits (α=0.05). Same letters indicate no significant difference at final harvest (α=0.05). C, Control; SSA, treated sewage sludge ash; STR, struvite containing P fertilizer.

BSs had no significant effect on the growing speed of the plants (**Supplementary Table 2**). All plants started growing at the same rate, but by the second measurement time, the STR and SSA treatments were significantly higher than the unfertilized control (**Figure 3B**). Likewise, BSs had no significant effect on leaf area and stem diameter. Differences among fertilizer treatments were minuscule at the start, while the SSA and STR started to diverge from the unfertilized control treatment by the second measurement time. At harvest, the SSA and STR treatments did not vary significantly from one another regarding any plant trait (**Figures 3A–D**).

**Figure 4:**
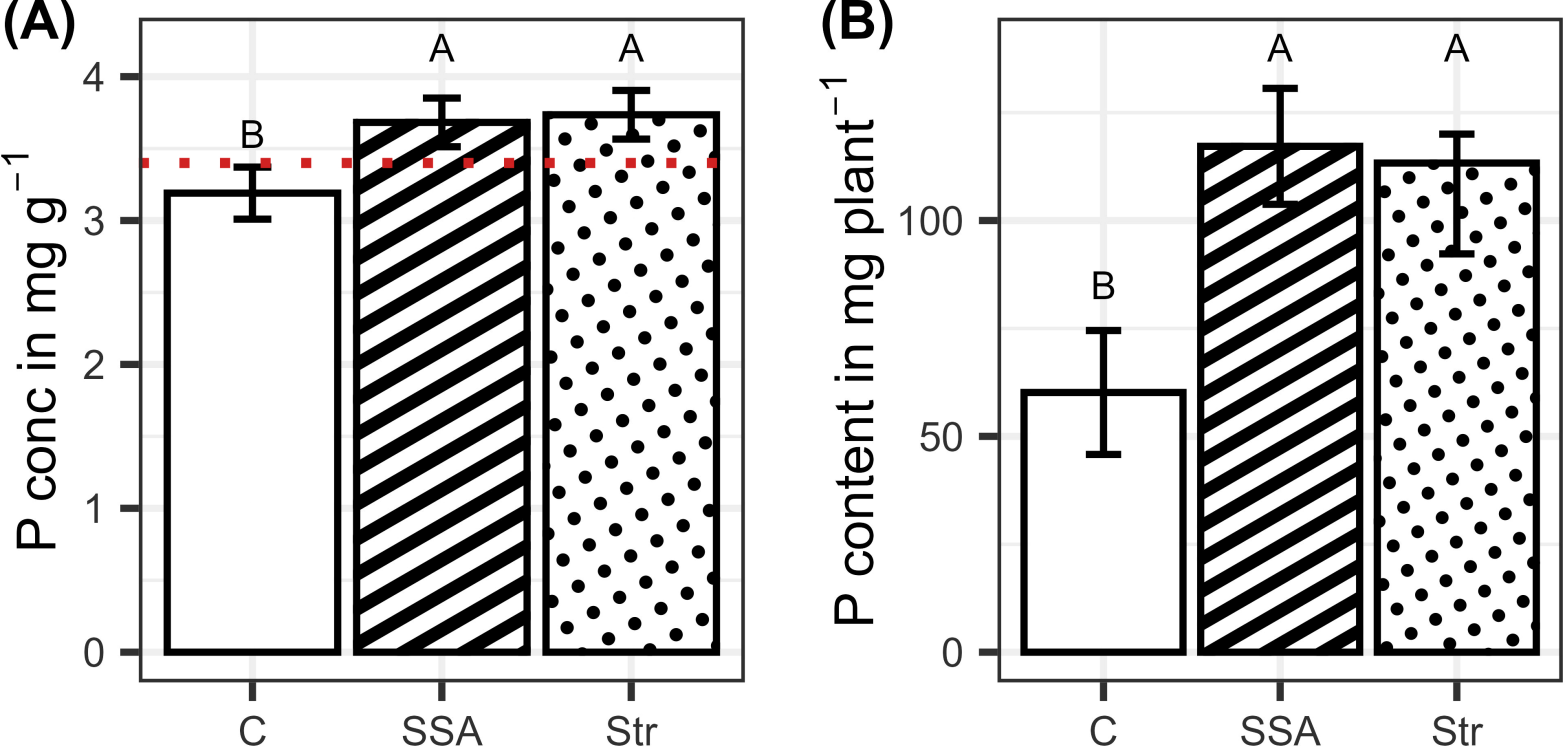
**(A)** P concentration and **(B)** P content in dependence on the fertilization (n=48). Means are displayed by bars with their confidence limits (α=0.05). In **(a)**, the red dotted line depicts the critical P concentration threshold underneath plants face P limitation. Same letters indicate no significant difference at final harvest (α=0.05). C, Control; SSA, treated sewage sludge ash; STR, struvite containing P fertilizer.

**Figure 5:**
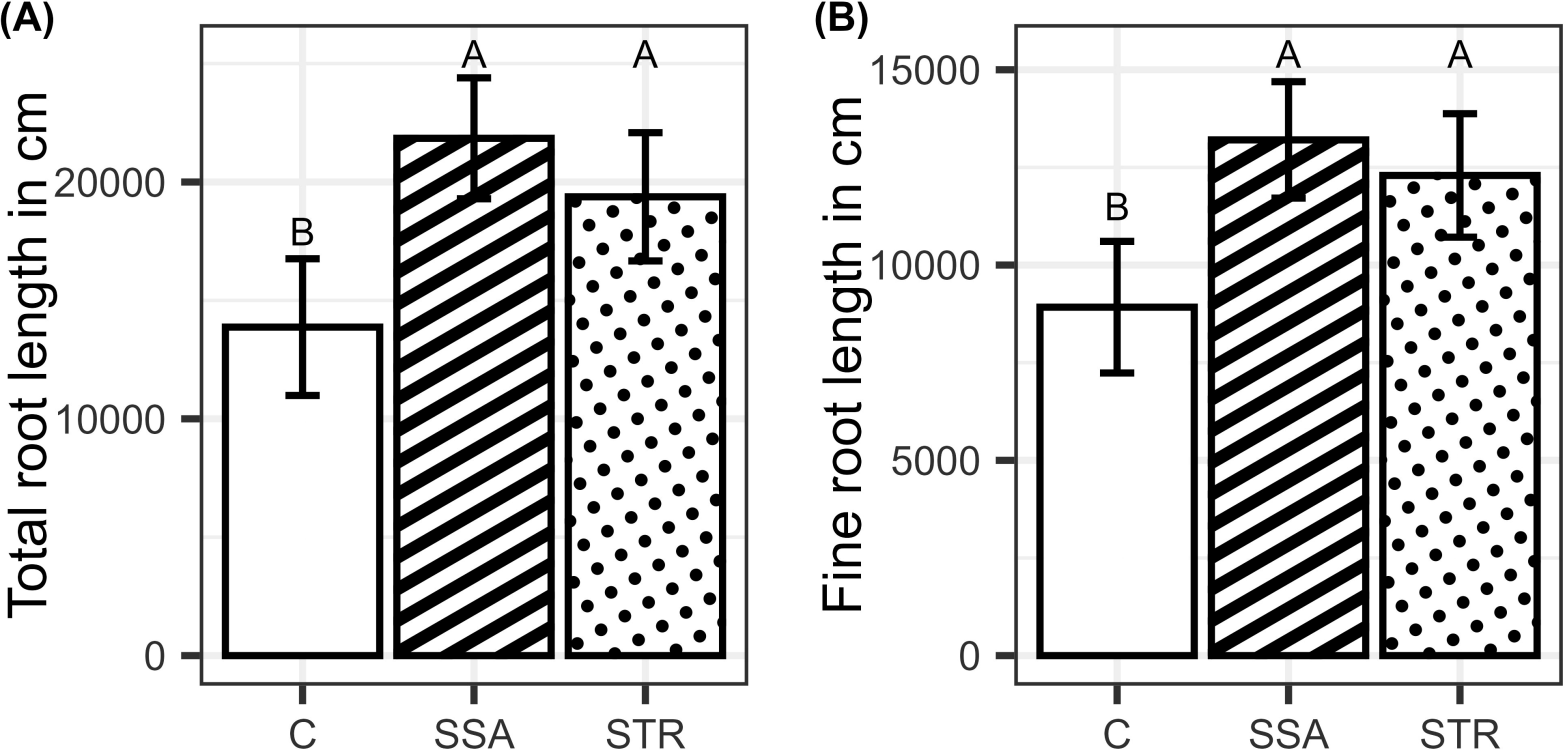
**(A)** Total root length and **(B)** fine root length in dependence on the fertilization (n=48). Means are displayed by bars with their confidence limits (α=0.05). Same letters indicate no significant difference at final harvest (α=0.05). C, Control; SSA, treated sewage sludge ash; STR, struvite containing P fertilizer.

Fertilizing the plants increased the P content and concentration of the plants, while the fertilizers did not differ significantly from each other (**Figures 4A, B**). BSs did not affect root growth significantly, and only the fertilization effect was visible. Consistently, the two fertilizers increased root growth compared to the control but did not differ from each other (**Figures 5A, B**).

**Figure 6:**
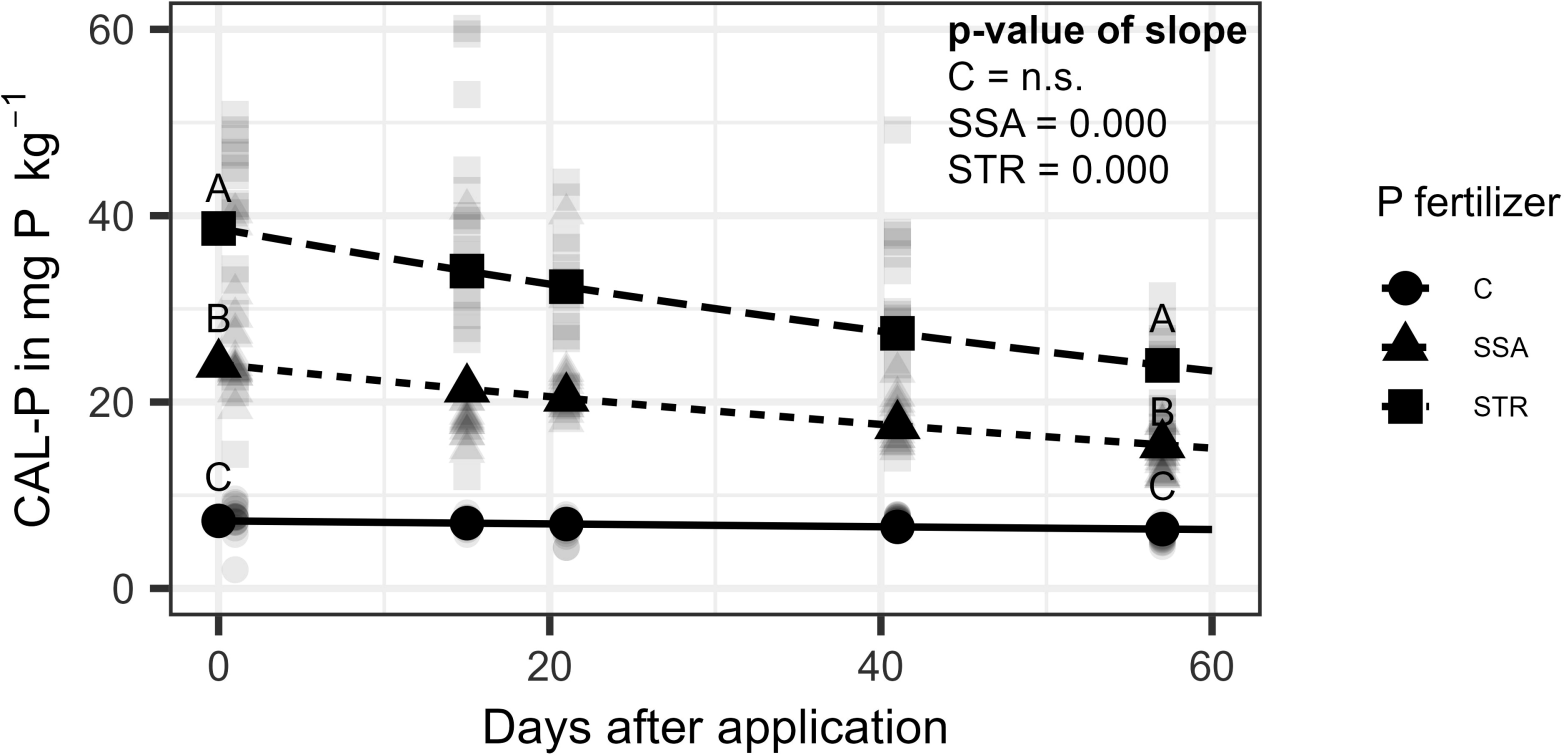
CAL-P content in dry soil as a function of time for the fertilization (n=240). Points represent the estimate from the model at the respective measuring time point while the lighter dots are raw measurements. Same letters indicate no significant difference between the fertilizers at the respective time point (α=0.05). C, Control; SSA, treated sewage sludge ash; STR, struvite containing P fertilizer. P-values of the slopes are shown in the upper right corner, which are significant for SSA and STR.

Concerning the soil analysis, CAL-P was not affected by the BS application, but by time and fertilization (**Supplementary Table 2**). At the beginning of the trial, all fertilization treatments varied significantly from each other with the STR treatment leading to the highest CAL-P content of 38.6 mg (kg dry soil)^-1^ followed by the SSA treatment with 24.1 mg (kg dry soil)^-1^ (**Figure 6**). The slopes of the two fertilizers are not significantly different from each other leading to a parallel decrease over time for the fertilizer treatments while the control stayed stable at the same level of around 7 mg (kg dry soil)^-1^.

**Figure 7:**
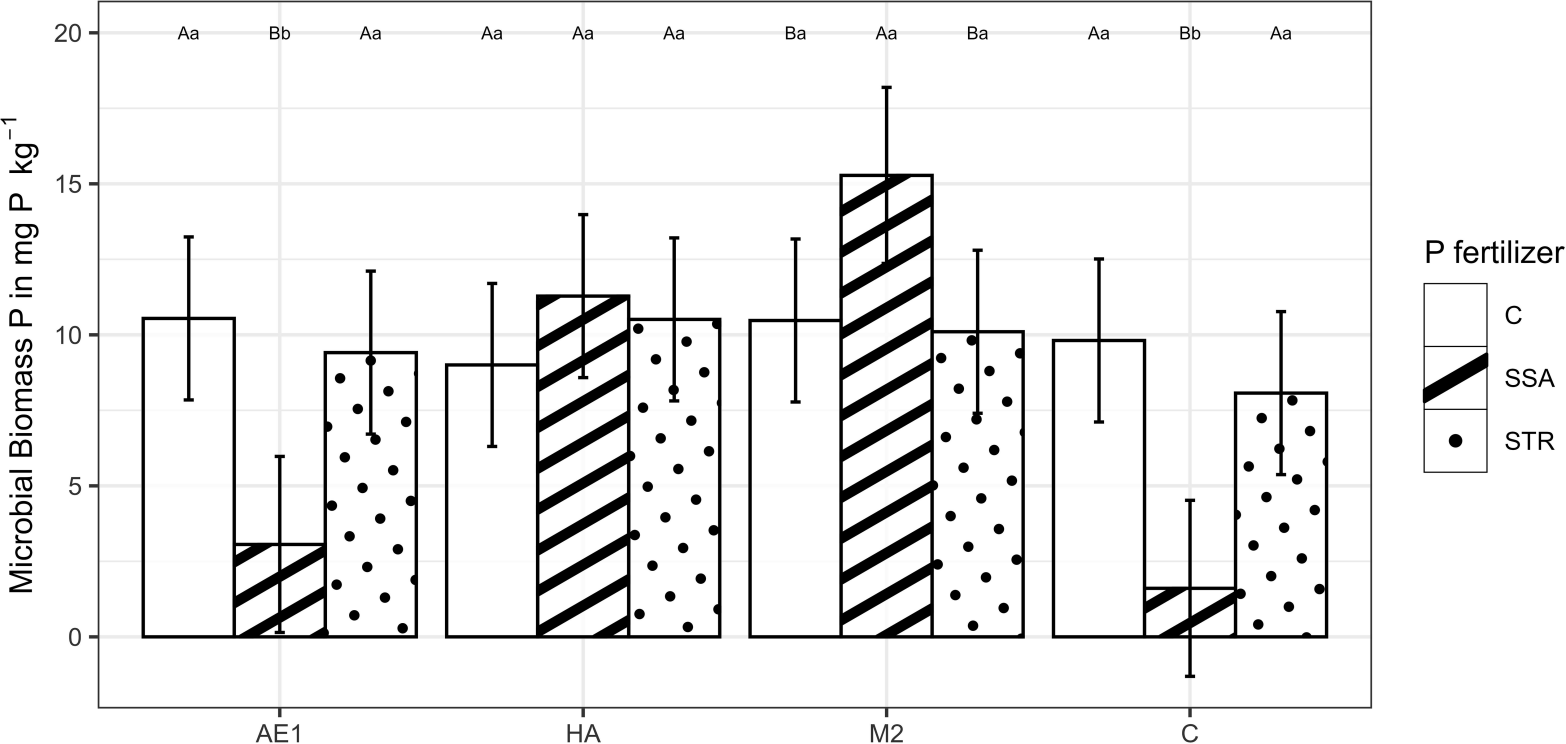
Microbial Biomass P in dry soil in dependence on biostimulants and fertilization (n=48). Means are displayed by bars with their confidence limits (α=0.05). Same capital letters indicate no significant difference of the fertilization within the BS treatment (α=0.05). Same small letters indicate no significant difference of the biostimulants within the fertilization (α=0.05). C, Control; SSA, treated sewage sludge ash; STR, struvite containing P fertilizer; AE1, plant extract; HA, humic acid; M2, microbial consortia.

The BSs had a statistically significant effect on microbial biomass P (**Supplementary Table 2**). The application of the SSA resulted in a considerable drop of the microbial biomass P in the control and extracts, which could not be observed with humic acids and microbial consortium (**Figure 7**). When the soil was inoculated with the BS “M2” the application of SSA led to an increase in microbial biomass P (15.3 mg P (kg dry soil)^-1^) compared to the other fertilization treatments (10.5 mg P (kg dry soil)^-1^ for the unfertilized control and 10.1 mg P (kg dry soil)^-1^ for the STR) (**Figure 7**). Regarding the enzyme activity, BSs and fertilization had no significant effect on the acidic or alkaline phosphatase activity (**Supplementary Table 2**).

The pH was significantly affected by BS application (p = 0.0157) and fertilizer treatment (p<0.0001). While the fertilizers slightly increased the pH by 0.2 from 4.9 to 5.1, the plant extract and the humic acid decreased the pH by 0.1 compared to the control (5.0), respectively (**Supplementary Table 4**).

## 4 Discussion

### 4.1 Fertilization and biostimulants impact on plant growth and nutrient uptake

BSs failed to ensure a promotion of plant growth under P limiting conditions with and without the supply of recycled fertilizer products. The reliability of BS product is limited due to their nature of indirectly influencing the soil which is also highlighted by existing studies in which BSs improved P fertilizer use efficiency of recycled fertilizers (Mpanga et al., 2018; Wollmann and Möller, 2018) and others in which they did not (Lekfeldt et al., 2016; Wollmann et al., 2018; Jastrzębska et al., 2019). The soil had a strong P limitation. Previous studies confirmed that a starter fertilization of P under strongly P-limiting conditions is needed to ensure or improve the action of various beneficial microorganisms such as AMF and P-solubilizing microorganisms (Treseder and Allen, 2002; Schütz et al., 2017) but also for active natural compounds such as humic acids and seaweed extracts (Li et al., 2022). However, the fertilization of P led to a significant increase in soil P turnover activity, P availability, and plant performance. The recycled P fertilizers nourished the plants sufficiently with P as P concentrations in shoots are above the critical threshold (Bergmann and Neubert, 1976) which may also explain the lack of action by the applied BSs.

Even though in the pot and incubation trial the two fertilizers differed clearly concerning their increase in the CAL-P content in soil, both can be considered equivalently effective regarding their impact on the plant growth parameters as well as nutrient uptake. Similar results were also obtained by previous studies (Nanzer et al., 2014; Severin et al., 2014; Wollmann et al., 2018) and it is widely recognized that CAL-P is not a direct predictor of plant growth and plant P uptake, especially when using alternative P sources (Duboc et al., 2017).

### 4.2 The impact of biostimulants and recycled fertilizers on soil P availability, P fertilizer availability, and soil P turnover

One main result regarding BS application to the soil is the higher CAL-P content in the case of the application of humic acids while other BSs did not influence the CAL-P content. Previous studies underlined this result by achieving a higher P availability when humic acids are applied together with fertilizers (Wang et al., 1995; Du et al., 2013). Humic acids can directly act against P adsorption onto minerals to the soil by competing with P for binding sites, by the dissolution of bound P, and by the inhibition of P precipitation (Du et al., 2013; Perassi and Borgnino, 2014; Gerke, 2021). Thus, these mechanisms can explain the correlation of the applied humic acid amount and the P availability in soil (Yuan et al., 2022). The application of humic acids may be more reliable than the inoculation of microorganisms which may not establish in soil or colonize the rhizosphere (Podile et al., 2014) or by an insufficient P solubilizing capability to deliver nutrients to the plants (Raymond et al., 2021). 2021). However, the impact of humic acids was not consistent in both soils in our study. This soil-dependent behavior was also observed in previous studies (Maluf et al., 2018) and could be related to soil pH (Gerke, 2021), clay contents (Maluf et al., 2018), and possibly other edaphic factors. Nevertheless, the effect of humic acids on CAL-P was possibly overshadowed in the pot trial by the effect of the plant and fertilizers. With higher amounts of applied humic substances, the impact on P mobilization could be further enforced and possibly be also relevant in plant trials like implemented in previous studies (Hua et al., 2008).

The microbial biomass P was not significantly altered without the plant in the incubation trial while a significant BS and SSA interaction was detected in the pot trial. This is an indicator of the necessity of a plant for the BSs to establish or interact with soil nutrients, especially for the microorganisms which need a carbon-rich environment to grow. In the pot trial, a sharp drop of microbial biomass P was observed when pots were fertilized with SSA. Previous studies have also detected a similar response when plants were fertilized with P (Clarholm, 1993) while in other studies microbial biomass P was positively affected by a P fertilization (Shi et al., 2020). However, it seems that humic acids and the microbial consortia have the capability to prevent such a decrease. Other studies revealed that seaweed extracts and humic acids shaped microbial community structure in soil but also on plant roots (Renaut et al., 2019; Yuan et al., 2022) and plant extracts could stimulate microbial degradation of organic material (Chen et al., 2002). Thus, also plant extracts can potentially alter the microbial community. Increasing the application frequency and the dosage of plant extracts may be a strategy to induce effects in microbial communities (Alam et al., 2013). The combination of the microbial consortia 2 and the SSA treatment even appeared to favor an establishment of the microbes while this was not the case for other fertilizers and microbe combinations. Despite this significant impact on the microbial biomass P, plant traits were not affected by the BS application.

Phosphatase activity in soil was not affected by BS application. In the contrary, previous studies have shown a reduction of phosphatase activity in soil by humic acids (Malcolm and Vaughan, 1979; Yuan et al., 2022) and a stimulation of phosphatase activity by mycorrhization of plants (Tarafdar and Marschner, 1994; Joner et al., 2000), by phosphate solubilizing bacteria (Kim et al., 1997) and by the addition of seaweed extracts (Chen et al., 2023). However, in none of the cases, the phosphatase activity was measured without plants. In our trial with plants, the influence on BSs may have been masked by a stronger influence of plants at harvest stage due to dense rooting of the pots. However, other studies also concluded that mobilization of P turnover processes in soil could not be identified as an important mechanism contributing to improved P availability by biostimulants, but rather the promotion of root growth (Raymond et al., 2021; Nkebiwe et al., 2024). In the incubation trial, we detected a fertilization effect on acid and alkaline phosphatase activity, while this was not recognizable in the pot trial. In the literature, consistency for fertilization effects on phosphatase activity and the correlation of soil P content is not given. For example, Garg and bahl (2008) found an increase in acid phosphatase activity with increasing soil P content, while Spiers and McGill (1979) concluded the opposite or could not find an impact of fertilization on phosphatase activity. In our case, it can be assumed that a lower P-availability in the soil itself increases the phosphatase activity potential in soil as plants and microorganisms require a higher mobilization capability with a lower P-availability. A short-term fertilization, however, can stimulate phosphatase activity in soil. Another decisive factor whether phosphatase activity is stimulated or not by P addition, is the P source provided via fertilization. Highly soluble P fertilizers decreased phosphatase activity while non-soluble P fertilizers did not affect or slightly increased the phosphatase activity (Redel et al., 2019). Both used recycled fertilizers are characterized by a low immediate solubility in soil, which could explain the stimulation of phosphatase activity by both fertilizers. As above mentioned, plants densely rooted the pots at the end of the trial, potentially masking an impact of the treatments, in the pot trial. Nevertheless, it can also be argued that available P content in soil is not a good predictor of phosphatase activity in soil. Margalef et al. (2017) could demonstrate that a correlation of acid phosphatase activity and available P content is not given, and that the enzyme activity is more connected to the available organic P content in soil.

## 5 Conclusion

Although BSs were able to influence P availability and microbial biomass P in the soil, the impact on plant growth was negligible in this trial. The outcomes of the analyses of the soil without plants and with plants did not correlate concerning the BSs highlighting the importance to prove BSs effect not only in *in-vitro* trials but in the actual system in which it shall be used in later stages. The fertilization increased plant growth, soil P turnover and available P content in soil in a higher magnitude than any BS effect. The treated sewage sludge ash resulted in an equivalent plant growth than the struvite containing P fertilizer even though the CAL-P was significantly lower. Thus, the assessment of new recycled fertilizers based on current extractive analyses for plant available P such as CAL-P extraction does not seem to be suitable, and plant trials are recommended to evaluate the replaceability of conventional synthetic fertilizers by these new products.

## Data Availability

The raw data supporting the conclusions of this article will be made available by the authors, without undue reservation.
