# Supplementary material for: Limitations of soil-applied non-microbial and microbial biostimulants in enhancing soil P turnover and recycled P fertilizer utilization - a study with and without plants
Source: Front Plant Sci. 2024 Nov 12;15:1465537. doi: 10.3389/fpls.2024.1465537 (PMC11588478; doi:10.3389/fpls.2024.1465537)
Supplement: Supplementary file 1 [file DataSheet1.docx]

Supplementary Table 1: ANOVA-Results of the linear mixed model. ns = not significant, * = p-value <0.05, ** = p-value<0.005, *** = p-value <0.0005. B = biostimulants, F = fertilizer, S = soil, T = time after fertilization. X means that the term was not included in the model of this response variable.

|  | CAL-P | Microbial Biomass P | Acid Phosphatase Activity | Alkaline Phosphatase Activity |
| --- | --- | --- | --- | --- |
| B | ** | ns | ns | ns |
| F | *** | ns | * | * |
| S | *** | X | *** | *** |
| T | *** | ns | X | X |
| BxF | ns | ns | ns | ns |
| BxS | ns | X | ns | ns |
| BxT | ns | ns | X | X |
| FxS | *** | X | ns | ns |
| FxT | *** | ns | X | X |
| BxFxS | * | X | ns | ns |
| BxFxT | ns | ns | X | X |
| BxSxT | ns | X | X | X |
| FxSxT | *** | X | X | X |
| BxFxSxT | ns | X | X | X |

Supplementary Table 2: ANOVA-Results of the linear mixed model. ns = not significant, * = p-value <0.05, ** = p-value<0.005, *** = p-value <0.0005. B = biostimulants, F = fertilizer, T = time after fertilization. X means that the term was not included in the model of this response variable.

|  | Plant traits measured several times during the growing period | Plant traits measured once at harvest | CAL-P | Microbial Biomass P | Acid Phosphatase Activity | Alkaline Phosphatase Activity | pH |
| --- | --- | --- | --- | --- | --- | --- | --- |
| B | ns | ns | ns | ** | ns | ns | * |
| F | *** | *** | *** | *** | ns | ns | *** |
| T | *** | X | *** | X | X | X | X |
| BxF | ns | ns | ns | ** | ns | ns | ns |
| BxT | ns | X | ns | X | X | X | X |
| FxT | *** | X | *** | X | X | X | X |
| BxFxT | ns | X | ns | X | X | X | X |


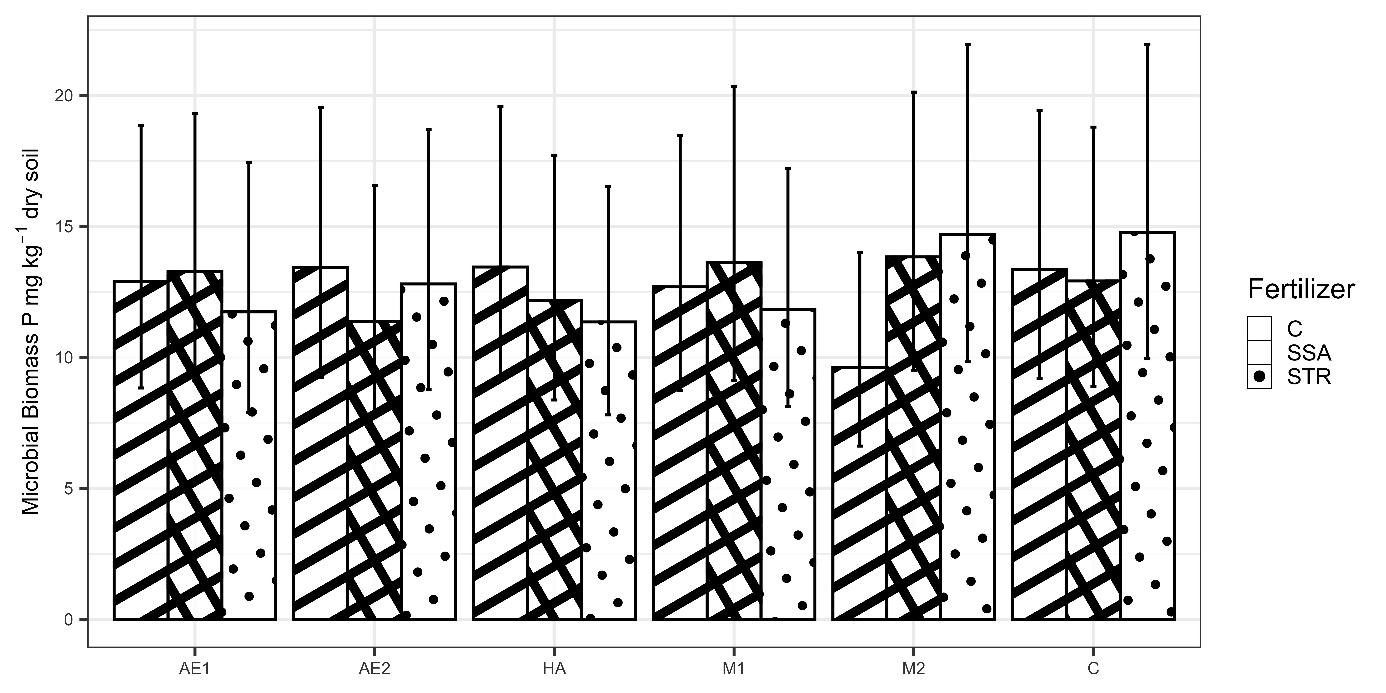


Supplementary Fig 1: Microbial Biomass P in dry soil in dependence on biostimulants and fertilization (n=180). Medians are displayed by bars with their confidence limits (α=0.05). C = Control, SSA = treated sewage sludge ash, STR = struvite containing P fertilizer, AE1+2 = plant extract, HA = humic acid, M1+2 = microbial consortia, C=control.

Supplementary Table 3: pH value in dependence on biostimulants, soil and fertilizer. AE1+2 = plant extract, HA = humic acid, M1+2 = microbial consortia. C = Control, SSA = treated sewage sludge ash, STR = struvite containing P fertilizer.

| Biostimulant | Soil | Fertilizer | pH |
| --- | --- | --- | --- |
| AE1 |  |  | 5.3 |
| AE2 |  |  | 5.3 |
| HA |  |  | 5.3 |
| M1 |  |  | 5.3 |
| M2 |  |  | 5.3 |
| C |  |  | 5.3 |
|  | Class B | C | 5.6 |
|  | Class B | SSA | 5.7 |
|  | Class B | STR | 5.7 |
|  | Class A | C | 4.8 |
|  | Class A | SSA | 4.9 |
|  | Class A | STR | 4.9 |

Supplementary Table 4: pH value in dependence on biostimulants, soil and fertilizer. AE1 = plant extract, HA = humic acid, M2 = microbial consortia. C = Control, SSA = treated sewage sludge ash, STR = struvite containing P fertilizer.

| Biostimulant | Fertilizer | pH |
| --- | --- | --- |
| AE1 |  | 5.0 |
| HA |  | 5.0 |
| M2 |  | 5.0 |
| C |  | 5.0 |
|  | C | 4.9 |
|  | SSA | 5.1 |
|  | STR | 5.0 |
